# Echocardiography in Left Ventricular Assist Device

**DOI:** 10.4103/1995-705X.73222

**Published:** 2010

**Authors:** Sherif M. Helmy, Alia Albinali, Rachel Hajar

**Affiliations:** Echocardiography Laboratory, Cardiology and Cardiothoracic Surgery Department, Hamad Medical Corporation, Doha, Qatar

**Keywords:** Left Ventricular Assist Device, Heart Failure, Surgery, Echocardiography

## Abstract

LVAD = Left Ventricular Assist Device; LV = Left Ventricle; LA = Left Atrium; AO = Aorta

A 41-year-old male Indian patient was admitted to our hospital due to refractory congestive heart failure. The patient received a left ventricular assist device (LVAD) as destination therapy. The images show the preoperative, intraoperative, and postoperative echocardiography findings [Figures 1–5].

**Figure 1:**
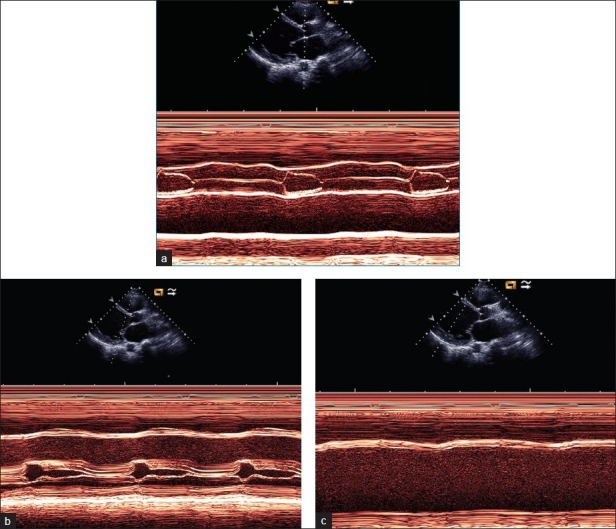
Preoperative M mode study showing a low cardiac output pattern of the (a) Preoperative M-mode study showing a low cardiac output pattern of the aortic valve, (b) Low output pattern of mitral valve, and (c) Dilated left ventricle (LV) with low output pattern

**Figure 2:**
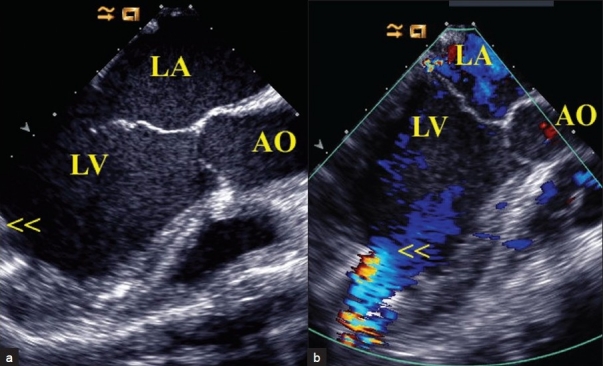
(a) Intraoperative tranesophageal echocardiography at 115° showing the LVAD inflow conduit from the LV apex (arrows) and (b) the color flow in the conduit (arrows)

**Figure 3:**
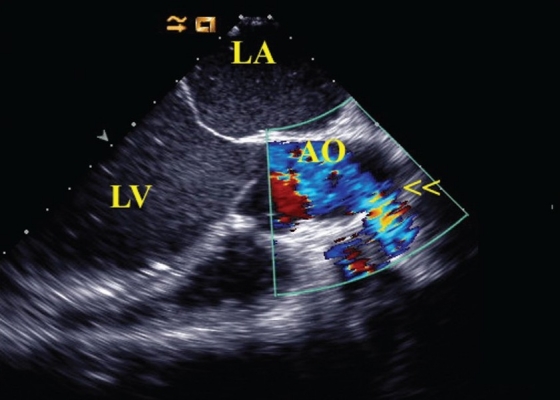
Intraoperative transesophageal echocardiography at 115°, showing the outflow conduit of the LVAD into the ascending aorta (arrows)

**Figure 4:**
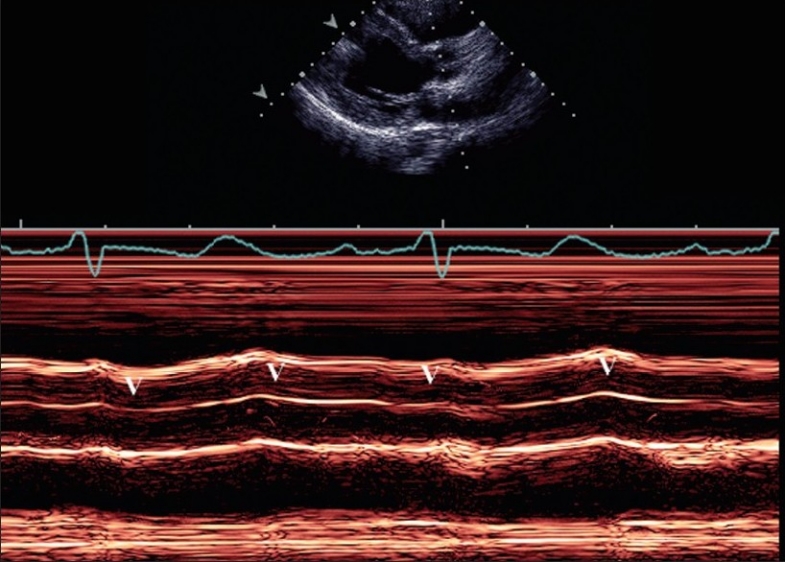
Post-LVAD implantation M mode echocardiogram of the aortic valve and left atrium shows closed aortic cusps throughout systole and diastole (v), denoting the well-functioning LVAD

**Figure 5:**
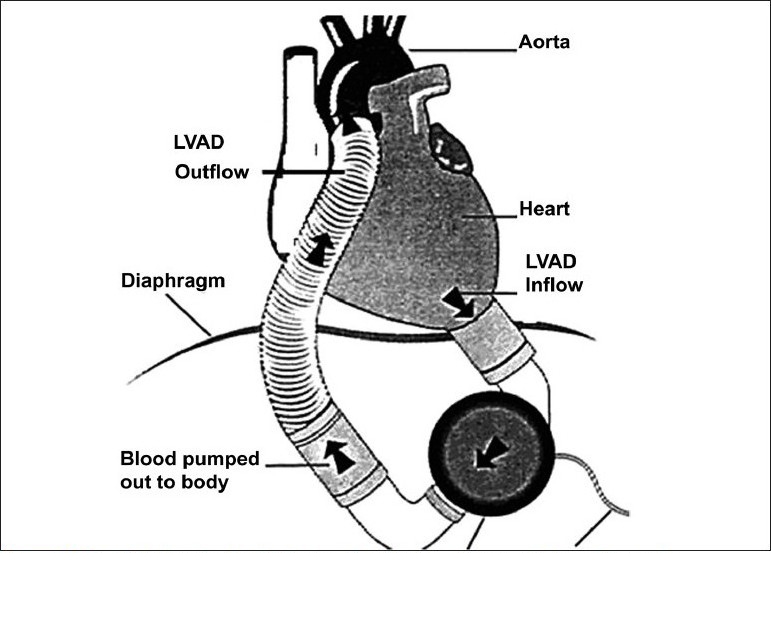
Diagram showing the LVAD and its inflow and outflow conduits.

